# Mapping the brain's metaphor circuitry: metaphorical thought in everyday reason

**DOI:** 10.3389/fnhum.2014.00958

**Published:** 2014-12-16

**Authors:** George Lakoff

**Affiliations:** Department of Linguistics, University of California BerkeleyBerkeley, CA, USA

**Keywords:** conceptual metaphor theory, neural circuitry, embodied cognition

## Abstract

An overview of the basics of metaphorical thought and language from the perspective of Neurocognition, the integrated interdisciplinary study of how conceptual thought and language work in the brain. The paper outlines a theory of metaphor circuitry and discusses how everyday reason makes use of embodied metaphor circuitry.

## How we got here: conceptual metaphor in everyday reason

The discovery of conceptual metaphor independently by Michael Reddy and myself in the late 1970's showed that metaphor is primarily conceptual, and secondarily linguistic, gestural, and visual (Reddy, 1979; Lakoff and Johnson, 1980/2002). There are metaphorical ideas everywhere and they affect how we act. Metaphorical thought and the metaphorical understanding of situations arises independent of language. This discovery led almost immediately to the hypothesis that everyday reason that is understood as “abstract” (not just about “concrete” physical objects and actions) make use of embodied metaphorical thought (Lakoff and Johnson, 1999).

Reddy had found that the abstract concepts of communication and ideas are understood via a conceptual metaphor:

- Ideas Are Objects.
- Language Is a Container for Idea-Objects.
- Communication Is Sending Idea-Objects in Language-Containers.

This notation from Lakoff and Johnson characterizes a conceptual mapping from a “source domain” frame for sending objects in containers to a “target domain” frame for communicating ideas via language.

Reddy found over 100 classes of expressions for this metaphor. Examples include: *You finally* ***got through to*** *him. The meaning is right there* ***in the words***. ***Put*** *your thoughts* ***into*** *clear language. Your words are* ***hollow***. And many more. His point was that the generalization covering the linguistic metaphors was not in language, but in the metaphorical concept of communication as sending idea-objects in language-containers.

Reddy furthermore pointed out that the metaphor created an important inference about communication: the speaker is primarily responsible for its success. If you put an object in a container and send it, the receiver will find the *same* object inside. Reddy observes that in real communication, the hearer has as much responsibility as the speaker, and that what the hearer hears is very often not what the speaker intends. However, the metaphor is often taken literally, as it were true.

### Metaphor systems and domains of thought

A crucial idea in the study of metaphor is the *conceptual metaphor system* for characterizing a *domain of thought*. This idea was first worked out by Eve Sweetser and Alan Schwartz (see Lakoff and Johnson, 1999, chapter 12). They observed that there is a domain of Mind (a metaphorical target) that is understood via a very general metaphor that is in turn split into four subcases, each associated with a separate source domain. The general metaphor is the follow conceptual mapping:

- The Mind Is a Body.
- Mental Functioning Is Bodily Functioning.
- Ideas Are Objects of Bodily Functioning.

The four special case conceptual metaphors are:

- Thinking Is Moving; Ideas are Locations; Communicating is Leading; Understanding is Following.
- Understanding is Seeing; Ideas are Things Seen; Communication Is Showing.
- Thinking Is Object Manipulation; Ideas Are Objects; Communication Is Sending; Understanding is Grasping.
- Thinking Is Eating; Ideas Are Food; Communication is Feeding; Understanding is Digesting.

There are many linguistic examples of each.

- Moving: *reach a conclusion; go off on a tangent; do you follow me; go step-by-step, …*
- Seeing: *see what I mean; point of view; shed light on; clear; brilliant, …*
- Manipulating: *turn it over in your mind; toss ideas around; I gave him that idea;…*
- Eating: *food for thought; raw facts; half-baked ideas; digest; He won't swallow that; …*

What seems to define these domains are embodied brain regions or structures significantly involved in performing these functions. The questions raised by this analysis are, What is a domain in the brain? What defines a metaphor system and a domain neurally?

Domains seem to be characterized by hierarchically structured frames. A frame is a complex schema, a mental structure that organizes knowledge. Each frame makes use of primitive concepts and may make use of conceptual metaphors. The elements of a frame are called Semantic Roles.

For example, the semantic roles of the Seeing Frame are: The Viewpoint, The Viewer, Eyes, Light, The Directing of the Eyes, The Act of Seeing, Things Seen, The Gaze (the line from the eyes to the thing seen); Degree of Clarity. There is also knowledge about seeing: You need enough light to see; light has a source; the gaze must extend from the eyes to the thing seen in order to see; things look different from different viewpoints; and so on.

A crucial thing we learn from this is that important abstract concepts are not merely understood via one conceptual metaphor, but via multiple conceptual metaphors that provide different understandings of the concepts. For example, Communication is not just Sending, but it is also Leading (when Thinking is Moving), Showing (when Understanding is Seeing Clearly), and Feeding (when Thinking is Eating). Ideas, metaphorically, can be not only Manipulable Objects, but Locations and Food as well.

Lakoff and Johnson (1999) have shown that important concepts like Event, Action, Causation, the Mind, the Self, Morality, and Being are each defined via multiple conceptual metaphors, sometimes between a dozen and two dozen.

I made a discovery similar to Reddy's at about the same time. I had found that the abstract concept of Love is commonly understood in terms of a Journey. There are lots of linguistic expressions of this sort: *Our relationship hit a dead-end street. The marriage is on the rocks. We're getting nowhere in this relationship. We're going in different directions. We're at a crossroads in our relationship. We're spinning our wheels in this relationship*. And many more. The generalization over these cases is not in the linguistic expressions but in a conceptual mapping (indicated by “==>”).

- Travelers ==> Lovers.
- Vehicle ==> Relationship.
- Common Destinations ==> Common Life Goals.
- Impediments to Travel ==> Relationship Difficulties.

### Embodied primary metaphors

Mark Johnson and I later discovered that this complex metaphor was made up of more basic components. There are primitive metaphors that are acquired in ordinary daily life when two basic embodied experiences regularly occur together. For example, purposes are understood as destinations. In everyday life, achieving purposes often requires getting to a destination. If you want a cold beer, you'll have to go to the refrigerator. In American culture, people are expected to have goals in life, and a couple in a long-term love relationship is expected to have compatible life goals. Metaphorically that means having common destinations. A relationship is a metaphorical vehicle for three reasons: First, a vehicle is a means of getting to a destination. Second, a vehicle is a *container*. In general, relationships are understood in terms of containers: you are *in* relationship; you can *enter* or *leave* a relationship. Third, intimacy is understood metaphorically in terms of closeness: *We're very close; we're drifting apart*. Thus, a relationship is conceptualized as a container in which you are close and which is a means for reaching destinations.

Johnson and I reasoned as follows: Why is intimacy metaphorized as closeness? Because intimacy requires being physically close. Why is a relationship a container? Because when you are growing up, you tend to live in the same enclosed space as your relatives. Purposes are conceptualized as destinations because, over and over again, to achieve a purpose you have to go to a specific location. The general principle is that regular correlations in real-world embodied experience leads to primitive conceptual metaphors—*embodied primary metaphors using embodied promitive concepts*—that can combine to form complex conceptual metaphors, like the Live Is a Journey metaphor.

These considerations led directly to the theory of embodied cognition. The most popular theory of meaning at the time was that concepts were all literal, that there were no metaphorical concepts, and that concepts got their meaning via truth conditions—directly from conditions holding objectively in the real world, independent of the intervention of human minds and brains. The existence of conceptual metaphors did not fit that theory. The idea that there are primitive conceptual metaphors that arise from regular correlations in embodied experience did not fit that theory. If we were right, then a new theory of meaning for concepts was necessary.

The most obvious candidate was a theory of embodied cognition. Physical concepts, like running and jumping, chairs and people, could be understood through the sensorimotor system: they can be performed, seen, felt. If abstract concepts get their meaning via conceptual metaphor, and if complex conceptual metaphors are made up of primitive conceptual metaphors that get their meaning via embodied experience, then the meaning of concepts comes through embodied cognition.

If that was so, Johnson and I realized that there should be significant real-world consequences. Take the metaphor of Labor as a Resource, where companies seek *cheap labor*, with workers seen as interchangeable commodities to be purchased for minimum cost in a *labor market* and working people are hired though the “Human Resource Department.” Thus, corporations, to maximize profits, should seek to minimize the “cost” of labor—by cutting pay and benefits, outsourcing, and laying off workers whenever possible. Johnson and I saw enormous social and political consequences arising from abstract thought being characterized metaphorically.

### Metaphor and the meaning of idioms

The earliest examples we looked at took us to the study of idioms. The traditional theory held that idioms had arbitrary meanings. We discovered that the meanings of a huge range of idioms were anything but arbitrary. They made use of conceptual metaphors! But not in any obvious way.

The first one I looked at was: *We're spinning our wheels in this relationship*. It has a conventional image, with knowledge about the image: The wheels are on a car. The car is stuck with the wheels spinning (either in sand, or on ice, etc.). The car isn't moving. We're putting a lot of effort into getting it moving, but it won't move. We are frustrated.

The Love Is a Journey mapping applies to the conceptual knowledge about the image. The car (a vehicle) is the relationship, the travelers are lovers and they are not making progress toward common destinations (compatible life goals). They feel frustrated.

That is what it means to be spinning your wheels in a relationship. **The conceptual metaphor applies to knowledge about the image, yielding the meaning of the idiom!**

But although the Love Is a Journey metaphor applies systematically in understanding this idiom, the literal meanings of the words in the idiom (“spinning” and “wheels”) are not mapped by this metaphor. Those words activate a conventional mental image with associated knowledge commonplace in one's culture. There is a system of metaphors fixed in the mind that applies naturally, automatically, very quickly, and unconsciously to such knowledge, linking the knowledge of the image to the meaning of the idiom.

There are a huge number of idioms like this. Consider *The marriage is on the rocks*. The marriage (the relationship) is a boat (a vehicle). A boat on the rocks is not moving forward. The couple in the boat is not progressing toward their common destination (compatible life goals). The boat is likely to be harmed in some way. Even if it gets free of the rocks, it may not be able to continue on the journey. That is, even if the marriage survives, the couple may still split up. And when the boat hits the rocks, the passengers may be hurt physically. Given the metaphor that psychological harm is physical harm, the couple may be psychologically harmed by the incident.

If you have that image for the idiom and that knowledge about the image, then that is what the idiom means metaphorically. That same Love is a Journey metaphor, applying to a different image and knowledge, yields a different meaning.

These constitute a special class of idioms: they are both are imageable and metaphorical. New ones are being created all the time (Lakoff, 1987, case study 2).

A Note: Metaphorical mappings occur at a certain level of generalization. In the Love Is A Journey metaphor, the relationship is a generalized vehicle. There are special cases of vehicles: cars, boats, planes (*We may have to bail out*), rockets (*We've just taken off), trains* (*We're off the track*). It's important to recognize the general level of the conceptual metaphor. Encountering *The marriage is on the rocks*, you should not conclude that the conceptual metaphor is Love Is a Boat.

A caution: Not every speaker has the same image and knowledge. For example, some speakers understand “on the rocks” in terms of a scotch on the rocks image and the idiom will seem to them to have an arbitrary meaning. For them, the Love is a Journey metaphor does not apply, and idiom is not metaphorical. It works for them as if it were a single lexical item with an arbitrary meaning, that is, one that does follow from the language. For example, it may mean, “will probably get a divorce.”

On the other hand, the arbitrary meaning may use a different conceptual metaphor, as in *The couple will probably split up*, which uses the conceptual metaphor that a relationship is a single entity made up of two parts. “Splitting up” means the relationship comes apart and there is no longer a single entity.

When a neuroscientist is using an idiom in metaphor research where there is averaging over a number of subjects, it is important to make sure that all the subjects use the same metaphor in understanding the idiom. That is not easy to do. Moreover, the metaphor may apply systematically not to the words “spin” and “wheels,” or to the words “on” and “rocks,” but rather to the concepts in the way the image is understood—if it is understood at all!

Some idioms are completely arbitrary, that is, you cannot figure out the meaning from the words. Take “by and large.” It was originally a nautical term from the days of sailing ships. To sail “by” meant close by the wind, whereas to sail “large,” meant with the wind fully behind you filling the sails (making them large). If a ship sailed well both “by and large,” then it sailed well under most conditions. Via the commonplace metaphor that Action Is Motion, with sailing as a special case of motion, sailing by and large came metaphorically to mean action by and large, that is, under most conditions. With the complete loss of “by and large” in its nautical meaning, the meaning of “by and large” kept the meaning of “mostly” but the systematic metaphorical relationship to the words was lost.

Some neuroscientists choose to study idioms with body part names like *hand*, or words for what body parts do, like *kick* or *bite*. The point is to see if the relevant body part word activates the brain region in the topographic map of the body in the motor cortex. But such idioms vary in their degree of arbitrariness and directness. There is a commonplace conceptual metaphor, Control Is Control by the Hands. It occurs in the understanding of idioms like *It's in your* ***hands*** *now, He's got the whole world in his* ***hands***, *They* ***handed*** *over the company to the Mafia*. In these cases there is a relatively direct metaphorical connection between hands and control. But that particular metaphor is not present in the understanding of *He's an old* ***hand*** *at phonological analysis; Tax cuts are* ***hand****outs to the wealthy; Don't bite the* ***hand*** *that feeds you*.

The idiom *kick the bucket* has been used in some neuroscience experiments to see if there is activation in the foot region of the motor cortex. What would one expect? Not much. First, there is a lot of variation across speakers. For many speakers it is an arbitrary idiom, with the meaning of *kick* playing no role at all in the meaning. For some there is a weak mental image. Here is mine:

The bucket is upright. There is some but not much liquid in it. It is weakly kicked over and what liquid there is spills out, and it is empty and on its side after the kick. There is a common conceptual metaphor that seems to be applying here: Life is a Fluid in the Body, as in sentences like *The life drained out of him; He's full of life; He's brimming with life*. The spilling out of the fluid from the bucket means death. But since there was not much fluid in it in the first place, it suggests a particular kind of death—death when there is not much life left, as with an old person expected to die soon. You won't say *She kicked the bucket* of a child run over by a car or a young woman who died in childbirth.

Incidentally an image like mine appears in a prominent place in two popular movies. In *It's a Mad, Mad, Mad, Mad World*, Jimmy Durante plays an old man who dies of a heart attack on a mountain. As rigor mortis sets in, his leg goes out and kicks over a bucket that tumbles down the mountain. In *Young Frankenstein*, the man soon to become the monster dies and, in rigor mortis, kicks over a slop bucket at the edge of the bed. The kicking of the bucket is a comic way of indicating death, a visual pun in two slapstick movies.

But for many speakers, *kick the bucket* is an arbitrary idiom, with no mental image of kicking. Even in the best of cases, one shouldn't expect much by way of foot activation in the motor cortex. The kicking is only indirectly connected to the death, and then only via a conceptual metaphor that has nothing directly to do with kicking. In addition, the bucket may be a container, like the body, but that's a weak connection. And for most speakers, there is no connection at all.

The morals for neuroscientists: Be aware of what kind of idioms you are using in your experiments and what their cognitive analysis is. Always list the idioms you are using in any write-up of your experiment. And test your subjects for the images they may or may not associate with the idioms.

### Emotion metaphors

In the early 1980's, Zoltán Kövecses and I discovered that systems of emotion metaphors arise from the physiology of emotions (Lakoff, 1987; Kövecses, 2000, 2002). For example, Paul Ekman and his colleagues found that when one is angry, skin temperature rises, blood pressure increases, and there is interference with accurate visual perception and fine motor control (Ekman et al., 1983). That is why we get such linguistic metaphorical expressions as *boiling mad, He exploded, blind with rage, hopping mad*, and many more (Lakoff, 1987, Case Study 1), (Wilkowski et al., 2009).

Damasio (1996) has observed that such bodily experiences have correlates in the brain's somatosensory system which are registered and can be seen via neuroimaging in the ventromedial prefrontal cortex as “somatic markers” that play an important role indecision making. This raises the possibility that emotions *are constituted by* the bodily effects that are registered in brain during emotional experience. Thus, it would be natural for emotions to be metaphorically conceptualized as those bodily effects, as Kövecses and I observed. This accords with the theoretical model of Lindeman and Abramson (2008) of the causal mechanisms of depression. They hypothesize that “(a) the inability to alter events is conceptualized metaphorically as motor incapacity; (b) as part of this conceptualization, the experience of motor incapacity is mentally simulated; and (c) this simulation leads to both feelings of lethargy and peripheral physiological changes consistent with motor incapacity.”

These ideas, together with our emotion metaphor research, raises the possibility that one can get insight into emotional states via neuroscience and the study of linguistic metaphors for physical states.

### Metaphor science in language

A whole field of metaphor science developed after 1980, including research on the role of conceptual metaphor in grammar. The first major paper on construction grammar came out in 1987, a 100+ page study of There-constructions that demonstrated the importance of conceptual metaphor in grammar (Lakoff, 1987, Case study 3). Since then, Adele Goldberg and Ellen Dodge, in book-length studies, have demonstrated how conceptual metaphors work in grammatical constructions (Goldberg, 1995; Dodge, 2010). Following those insights, Karen Sullivan has since provided the first general theory of how conceptual metaphor structures grammatical constructions (Sullivan, 2007, 2013).

Why does research on metaphor in grammar matter for an understanding of abstract thought? Because that research appears to show that there is a bifurcation in the way conceptual metaphor works in abstract thought.

1. There is a language-independent system in which abstract thought is understood metaphorically.
2. Language uses this system and extends it to a huge new range of abstract thought via metaphor. In the lexicon, this works via radial categories of lexical meanings (Lakoff, 1987, Case study 2). In grammar, indefinitely large extension works via the metaphor-in-grammar principles discovered by Sullivan (2013). An outstanding introductory text on these matters is (Dancygier and Sweetser, 2014). Examples will be given below.

### Mapping metaphor circuitry

In 1988, Jerome Feldman and I set up the Neural Theory of Language group at the International Computer Science Institute at UC Berkeley. Its goal was to apply neural computation to results in cognitive linguistics and embodied cognition (Feldman, 2006).

In 1997, Srini Narayanan worked out a neural computational theory of metaphor in his dissertation (Narayanan, 1997) and has expanded on that work greatly since then (Feldman and Narayanan, 2004; Loenneker-Rodman and Narayanan, 2012). He and I have been working on a theory of the neural circuitry required for thought and language. The following is a discussion of the current status of our research as it applies to metaphor.

- First, a statement of the theory.
- Second, an account of primitive embodied concepts and primary metaphors, which we see as the building blocks of abstract thought.
- Third, examples of common complexes of primary metaphors, to provide a sense of how commonplace abstract ideas that are complex arise from combinations of metaphor primitives, and how primary metaphor form systems of abstract thought.
- Fourth, examples of how complex combinations of multiple linguistic metaphors create complex abstract meanings.
- And finally, how all this fits into the current theory of neural cascades linking thoughts and language that expresses those thoughts.

## Our current neural theory of metaphor

Our current theory begins with a basic observation: The division between concrete and abstract thought is based on what can be observed from the outside. Physical entities, properties, and activities are “concrete.” What is not visible is called “abstract:” emotions, purposes, ideas, and understandings of other non-visible things (freedom, time, social organization, systems of thought, and so on). From the perspective of the brain, each of those abstractions are physical, because all thought and understanding is physical, carried out by neural circuitry. That puts “concrete” and “abstract” ideas on the same basis in the brain. Where conceptual metaphor theorists saw conceptual metaphor as conceptualizing the abstract in terms of the concrete, neural metaphor theory linked neural circuitry to other neural circuitry, allowing for a uniform theory, as follows:

- The human brain is structured by thousands of embodied metaphor mapping circuits that create an extraordinary richness within the human conceptual system. They largely function unconsciously.
- These mapping circuits asymmetrically link distinct brain regions, allowing reasoning patterns from one brain region to apply to another brain region (Lakoff, 2009).
- Each circuit characterizes a different form of metaphorical thought. Though metaphorical in content, the circuits reflect a reality, namely, real correspondences in real-world physical and social experiences starting in infancy.
- Where the experiences are essentially the same across cultures, the metaphor mappings tend to be the same. They appear to be learned by experience via neural learning. The asymmetry of the mappings appears to arise via STDP—spike-timing dependent plasticity—from which metaphor sources and targets can be predicted.
- Simple metaphorical thought is learned prior to, and independent of language, and plays an important role in the shaping of grammatical form (Johnson, 1999).
- Complex metaphorical thought is formed via a neural binding mechanism, to be discussed below.
- Complex metaphorical thought shows up not just in language, but in gesture, imagery (paintings, movies, dance, etc.), in mathematics (Lakoff and Núñez, 2000), science (Brown, 2003; Nersessian, 2008), and in moral and political ideology. (Lakoff, 1996/2002, 2004, 2006).
- The compositional properties of language, not surprisingly, lead to an unbounded range of complex metaphorical thought expressed linguistically.
- In the theory of neural cascades proposed by Srini Narayanan and myself, bidirectional cascades of neural activation link complex form (most notably, linguistic form) to complex metaphorical meanings characterized via connections to and from many brain regions.
- Metaphorical inferences arise via the neural simulation of situations that are understood, at least in part, via the activation of metaphor mapping circuits characterizing how the situations to be simulated are understood.
- The compositional properties of language allow for an even greater unbounded range of complex metaphorical thought, but still understood via embodied primitive concepts and primary metaphors.

### Primitive concepts and primary metaphors

#### Primitive concepts

The research of Talmy (2000), Langacker (1987), Fillmore (1968), Narayanan (1997) has indicated that there are embodied primitive concepts that arise in all natural languages.

Primitive concepts are all embodied via brain circuitry linked to the body via the sensorimotor system (Regier, 1997). Motion, for example, is characterized both via **topographic maps** of the visual field in which activation moves across the visual map, coordinated with **executing circuitry** for moving the body from an initial location, through a course of motion, to a final location.

The embodiment circuitry for different primitive concepts makes use of different parts of brain, which are anatomically organized by links to the body. For example, topographic maps for motion are in region MT (or V5) in the occipito-temporal lobe, while sequentially operating circuitry for executing bodily movement occurs in the premotor and supplementary motor cortices.

We know from research on mirror neuron systems that there are premotor-parietal pathways for linking action with vision (and imagined vision) (Gallese et al., 1996; Rizzolatti et al., 1996; Gallese and Lakoff, 2005).

We (that is, researchers in embodied neurocognition) hypothesize that primitive concepts have a **schema structure** that mediates between embodiment circuitry and complex concepts that are expressed by linguistic structures in natural language.

Elementary schemas have a Part-whole structure, with the entire schema as the Whole and the Semantic Roles as the Parts. Examples of Primitive Schemas with their Semantic Roles include:

- Motion (with a Mover, Source, Path, Goal, and possible Impediment),
- Containment (with an Interior, Boundary, and Exterior),
- Forces (with a Forcer, ForcedEntity, ForceDirection, ForceAmount, ForceEvent, and Force Result),
- PurposefulActions (with Precondition, Beginning Act, Central Act, Check for Achievement of a Purpose, Finishing Act, Final State, Consequence),
- and many more.

From a neural perspective, the elements of a schema are neural ensembles (called “nodes”), linked together to form a “neural gestalt.” A neural gestalt is defined by very simple activation strengths and threshold conditions: each semantic role node, when activated, activates the whole schema node, which in turn activates all of its role nodes.

**Complex concepts** are formed by **neural binding circuits**, which bind together schemas in different parts of the brain. A simple example is the concept INTO, which brings together schemas for Motion and Containment: the Source of the Motion is bound to the Exterior of a bounded region and the Goal of the Motion is bound to the Interior of the bounded region.

A **Binding Circuit** links two semantic role nodes in different schemas in different locations. It has to meet certain conditions. The schemas have to be able to function independently or as a single complex schema. The bound nodes have to be taken as “identical;” that is, they have to indistinguishable in their firing patterns. Both conditions are accomplished as follows.

1. There are two-way neural connections between the nodes, so that the firing of each one leads to the firing of the other.
2. There is a node that functions as a “gate” modulating the synapses connecting the two nodes. When the gate is not firing, the circuit is shut off for lack of sufficient neurotransmitters in its synapses. When the gate is firing, it introduces sufficient neurotransmitters in the synapses to allow the binding circuit to fire in both directions, coordinating the firing the two nodes.

Binding circuits are the primary mechanism of neural composition forming complex concepts by binding nodes across diverse brain regions.

#### Primary metaphors

Primary metaphors (Grady, 1997) are circuits that map primitive neural schemas onto other primitive neural schemas. This occurs when those pairs of neural schemas are regularly activated together because of real-world experience.

Here is a commonplace example. It is a very common occurrence in everyday life that one has to go to a specific location in order to achieve a given purpose. If you want a cold beer, you have go to the refrigerator where the beer is kept. If you want to brush your teeth, you have to the bathroom where the toothbrush and toothpaste are kept. And so on, case after case, day after day. Even infants, to feel secure, have to crawl over to where their favorite toy animal or their blanket is lying. These experiences give rise to the primary metaphor Purposes Are Destinations, which is widespread around the world. It maps the Motion Schema onto the Purposeful Action Schema as follows:

- The Mover maps to the Actor.
- The Motion maps to the Action.
- The Motion Source maps to the Action Precondition.
- The Motion Goal maps to the Purpose.
- An Impediment to Motion maps to a Difficulty in achieving the Purpose.

Each of these is a submapping; the whole collection of mappings jointly constitutes the metaphor mapping.

This mapping reflects a real-world fact. In the repeated experiences of going to a location to achieve a purpose, the elements of the motion schema correspond to the elements of the purposeful action schema. That is, the Actor is the Mover, the Action is the Motion, and so on.

Each of these correspondences in experience has a reflex in the brain: the corresponding nodes occur in different brain regions, but they fire together. Here is our hypothesis:

- The nodes that regularly fire together strengthen (via Hebbian learning) with regular firing.
- The neural activation spreads out from each neuron along existing pathways, creating neural links that get stronger as regular firing continues. The spreading keeps extending and strengthening.
- Eventually a shortest pathway is reached and a circuit is formed linking the two nodes.
- That circuit *is* the metaphor.
- Along that pathway there will be neural connections going in opposite directions, creating pairs of neurons where the axon extensions of each forms synapses with the dendrites of the other.
- This creates the condition for STDP—Spike-timing dependent plasticity—in which the synapse of the neuron that regularly spikes first is strengthened in its direction and the synapse of the other, later-firing neuron is weakened.
- The result is an asymmetric activation pattern, with activation going from Source to Target.

What determines the direction of first spiking? The answer is simple: the direction from which most activation comes regularly. That will be the metaphorical Source.

When we look at examples, this explanation appears to hold. Here are some examples:

- **More is Up, Less is Down**. Examples: *Stock prices went up. Turn the radio down*. Here Verticality is the Source and Quantity is the Target. Why? Because the brain is always computing verticality (even when you are sleeping) but not always computing quantity. Thus, there is more activation regularly flowing from the Verticality region to the Quantity region, which will lead to first-spiking ion the Quantity to Verticality direction.
- **Affection is Warm; Disaffection Is Cold**. Examples: *He's a warm person. She's cold as ice*. Here Temperature is the Source domain, and Affection is the Target. Why? The brain is always computing temperature, but not always computing affection. Thus, there is more activation regularly flowing from the Temperature region to the Affection region, which will lead to first-spiking in the Temperature to Affection direction.
- **Purposes are Destinations**. Examples: *There's nothing standing in my way. I hit a roadblock on this project. He has reached his goal*. This, of course, another obvious example. Not all of out motions are purposeful. We do a lot of aimless moving. This the Motion Schema will be activated more than the Purposeful Action Schema, resulting in first-spiking occurring in the Motion to Action direction, predicting that Motion will spike first in its direction and so will be the metaphorical Source.

So far, this works for the many cases checked out. The result is that the Source and Targets of primary metaphors can be predicted by the STDP theory of neural learning, which is a truly remarkable result.

There are hundreds, if not thousands, of primary metaphors structuring our conceptual system. They are learned via neural learning mechanisms early in life, usually before language, just by functioning in the everyday world.

Each primary metaphor neurally maps one primitive schema onto another, creating an asymmetric circuit linking them. But each primitive schema can also occur independently of any metaphor circuitry. That means that the metaphor circuitry must be gated: normally the gates modulating the connecting synapses would not be firing above base rate; the metaphor circuit is turned on when the gates are turned on, emitting sufficient neurotransmitters to allow activation to flow.

Each submapping has a gate. In the whole mapping, the gates work together. How? The theory requires the submapping gates and the gate for the whole mapping to form a gestalt circuit. Activating any submapping activates the whole mapping, and activating the whole mapping activates each submapping. As before, gestalt circuits have easy-to-learn combinations of activation and threshold strengths.

### Embodied cognition: the experimental results

In Narayanan's theory of primary metaphor, the metaphors are neural circuits asymmetrically linking two brain regions, a source region to a target region, with inferences from the source region used in the target region. That means that the physical consequences of source domain activation will, via the metaphor circuitry, yield corresponding target domain activation. It follows that the activation of metaphor circuitry can prime target domain behavior, where “prime” means that it contributes neural activation that makes the behavior more likely. Here are some cases of conceptual metaphors and the confirming experiments, in which there is source domain brain activation connected via metaphor circuitry to target domain brain regions that govern target domain behavior.

- Metaphor: Psychological Pain is Physical Pain.
- Study: Singer et al. (2006).
- Effect: In physical pain, the bilateral anterior insula and the anterior cingulate were active. They were also active in observing the experience of pain in a loved one. But with a stranger, the pain reaction in the anterior insula is lower.
- Metaphors: Crime Is a Virus vs. Crime is a Beast.
- Study: Thibodeau and Boroditsky (2013).
- Effect: When crime was framed metaphorically as a virus, participants proposed investigating the root causes of the problem and treating the community by enacting social reform by, for instance, eradicating poverty and improving education. When crime was framed metaphorically as a beast, participants took a much more direct approach in their proposals: catching and jailing criminals and enacting harsher enforcement laws.
- Metaphor: Morality is Purity.
- Study: Zhong and Liljenquist (2006).
- Effect: Subjects were asked to recall either a moral or immoral act in their past. Afterward, as a token of appreciation, the experimenters offered the subjects a choice between the gift of a pencil or of a package of antiseptic wipes. Those who had described an immoral act were more likely to choose the wipes. In a similar study later, subjects either did or did not have the opportunity to clean their hands. Those who were able to wash were less likely to respond to a request for help (that the experimenters had set up) that came shortly afterward. That is, washing expunged the guilt, and they saw no need to perform a helping act to expunge their guilt.
- Metaphor: Achieving a Purpose (or Desire) Is Reaching a Destination.
- Study: Harmon-Jones et al. (2011).
- Effect: “Leaning embodies desire: Evidence that leaning forward increases relative left frontal cortical activation to appetitive stimuli.”
- Metaphor: Affection Is Warmth.
- Study: Williams and Bargh (2008).
- Effect: Subjects holding warm coffee in advance were more likely to evaluate an imaginary individual as warm and friendly than those holding cold coffee.
- Metaphor: Affection Is Warmth.
- Study: Zhong and Leonardelli (2008).
- Effect: Subjects were asked to remember a time when they were either socially accepted or socially snubbed. Those with warm memories of acceptance judged the room to be 5 degrees warmer on the average than those who remembered being coldly snubbed.
- Metaphor: Important Is Heavy.
- Study: Zhong and Liljenquist (2006).
- Effect: Students told that that a particular book was important judged it to be physically heavier than a book that they were told was unimportant.
- Metaphor: Important Is Heavy.
- Study: Jostmann et al. (2009).
- Effect: Subjects with the heavy clipboards were more likely to judge currency to be more valuable and their opinions and their leaders more important than those with light clipboards.

Why does this happen? Conceptual metaphors are asymmetrical physical circuits in the brain allowing the consequences of source domain activation to apply in the cases of target domain activation. Those consequences can be a sense of filth after immoral behavior, inferences affecting crime policy, feelings of pain in empathy with a loved one, leaning forward physically, judgments of importance or temperature, and so on.

Experimental results of this sort were predicted by the idea of embodied conceptual metaphor. The experimental confirmation goes well beyond the cases just listed. The following two dozen studies will provide a sense of how robust the phenomenon is: Fishy smells induce suspicion, negative moral evaluation lessens the value of money, wiping the slate clean allows one to ignore past mistakes, unburdening yourself of a secret lowers the estimation of the upward slant of hills, and many more cases where metaphor circuitry linking two brain areas leads to behavior deriving from the physical metaphor linkage. Enjoy these: (Boroditsky, 2000; Singer et al., 2004, 2006; Aziz-Zadeh et al., 2006; Gibbs, 2006; Wilson and Gibbs, 2007; Casasanto, 2008; Boulenger et al., 2009; IJzerman and Semin, 2009; Schubert and Koole, 2009; Landau et al., 2010; Sapolsky, 2010; Desai et al., 2011; Lee and Schwarz, 2011, 2012; Saygin et al., 2011; Fay and Maner, 2012; Mattingly and Lewandowski, 2013; Pitts et al., 2013; Deckman et al., 2014; Galinsky et al., 2014; Knowles et al., 2014; Masicampo and Ambady, 2014; Sassenrath et al., 2014; Schoel et al., 2014; Slepian et al., 2014; Stellar and Willer, 2014).

### The neural metaphor system

This should not be thought of as a mere laundry list of cases. What links then together are the mechanisms that create the neural metaphor system—the neural learning mechanisms, the mapping circuits, the bindings, and the best-fit condition. “Best-fit” is more accurately called the conservation of energy law, namely, maximize the activation of existing circuitry with strong synapses that takes the least energy. Why, for example, should smelling fishy be behaviorally connected to suspicion (Lee and Schwarz, 2012)? The metaphor system contains all of the following. Note that special cases are instances of neural bindings of special to general cases that have been learned.

- Morality is Purity, Immorality is Rottenness
  *    Experiential basis:* In eating, pure food correlates with well-being, rotten food, with ill-being (Lakoff, 2008, Ch. 4)
- Thinking is Bodily Functioning.
  *    Special cases:* Communication is Sending;
  Thinking Is Eating: Understanding Is Digesting
  Understanding Is Perceiving: special case: Smelling
- Achieving a Purpose is Acquiring a Desired Object (Lakoff and Johnson, 1999, Ch. 11)
  *    Special Case:* Achieving a Purpose is Getting Desirable food
              A Difficulty Is Getting Undesirable food
  *                Special Case:* Rotten food
  *                  Special Case*: Rotten fish
- *Definition:* Suspicion is an understanding that someone has acted immorally to thwart someone else's purposes without their knowledge.

Here we have primary metaphors with special cases. They fit together to form a fixed complex metaphor system that defines the abstract concept of suspicion. Because this is an existing complex neural metaphor system in the brain, it can be activated in experiments to prime behavior. That is what is going on in embodied cognition experiments that show metaphor influencing behavior.

What is particularly interesting in the Lee and Schwarz paper is what they call “bidirectionality” in “metaphorical effects” in the experiments. They showed not just that fishy smells induce suspicion, but that *by inducing suspicion in subjects, that subjects were better able to distinguish the smell of fish oil from other smelly oils*. Their point is that, while other experiments show unidirectional, source to target metaphorical effects in experiments, they could produce bidirectional experimental effects.

What does this mean? Bear in mind that bidirectionality of *experimental effect* may or may not mean bidirectionality of the *metaphorical mapping*.

There are two important considerations not discussed by the experimenters. First, in Narayanan's neural theory of conceptual metaphor, STDP (spike-timing dependent plasticity) changes bidirectional ordinary Hebbian circuitry by strengthening synapses in the regularly first-spiking direction and weakening synapses in the opposite direction. Strengthening and weakening produces *relative* asymmetry, not absolute asymmetry. Moreover, the *amount* of strengthening and weakening depends on *how regularly* there is spiking in one direction rather than the other. In short, there should be variation in degree. Weakening does not mean no activation in that direction, only less, often much less. But it may still be enough to produce priming effects.

Narayanan's STDP theory makes the following prediction: Activating a conceptual domain that is a metaphorical target of one or more conceptual metaphors will provide some (often little) activation of one or more source domains of various metaphors. That is, a target domain can prime (to some extent, perhaps small) possible sources. For example, divorce should prime the splitting apart, going in separate directions. Difficulties should prime burdens, roadblocks, containment, uphill climbs, etc. Success should prime climbing ladders, getting fruit, reaching destinations, and so on. Cognitive linguists studying metaphor have long noticed such effects intuitively.

Second, there is the issue of language in general: the relation between words and their meaning *is* bidirectional. This is especially true of idioms that are both imageable and metaphorical. *Smell fishy* is such an idiom. It has an olfactory image. One can imagine what rotten fish (or fish oil) smells like. This could account for the bidirectional effect of the experiment, as follows.

Suspicion activates the idea of the *immoral* thwarting of someone's *purposes*. Immorality weakly primes rottenness (one of the primary metaphorical sources), and purposefulness weakly primes getting food to eat (one of the primary metaphorical sources), which in turn would thwart eating. Rotten food has the special case of smelly fish, and that smell image primes the idiom. That weak priming may still be strong enough to help distinguish fish oil smells from other smells.

Moreover, these are not mutually exclusive and the effects could combine in the experiment to yield a bidirectional experimental effect. The Lee and Schwarz experiment is lovely and points to the need to better understand the difference between unidirectionality in metaphorical mapping and unidirectionality in experimental effect.

### Metaphorical inference: the invariance hypothesis

How can conceptual metaphors provide content to abstract concepts, and how can different conceptual metaphors for a concept provide different content?

The circuitry constituting primary metaphors makes use of the structure of the source concept to reason about the target concept (Lakoff, 1993). For example, consider States, e.g., depression, confusion, etc. A State is understood metaphorically as a container, that is, a bounded region in space. Just as you can be in a bounded region, you can be in a state, just as you can enter a bounded region, you can enter a state, just as you can get out of a bounded region, you can get out of a state. The concept of a bounded region is used in the mapping from space to states. Or consider an executing schema that carries out a process. If you are building a house, the house is not yet finished. If you are metaphorically building an institution, the institution is still not complete.

Metaphor mappings are many-to-many.

- A target can have many sources. For example, Anger can be seen as Heat (boiling mad, all burned up, seething), Pressure (He exploded), Madness (go crazy, an insane rage), A Wild Animal (bristling with anger, unleashed his anger, a ferocious temper), and so on.
- Many targets can have the same source. Thus, More is Up, Good is Up, Happy is Up, and so on.
- The same linguistic metaphor can express conceptual metaphors with opposite meanings. For example, *It's downhill from here* can be an instance of Good is Up; Bad is Down and mean that things are getting worse. Or it can be instance of Action is Motion and Ease of Action is Downhill Motion, and can mean that things are getting easier. Another example is *Let's move the meeting ahead*. If Time is conceptualized as moving toward you, front to back, then the meeting is to be moved back in time, toward the past. If time is conceptualized as a landscape that you move over, then the meeting is to be moved to the future.

#### Embodiment and meaningfulness

Primitive concepts and primary metaphors are at the heart of any neural theory of concepts. The reason is that they are all embodied, and embodiment is what makes concepts meaningful, linking what is going on in our brains to our understanding of the real world.

That does not mean that we understand the real world as it is in some objective sense. But it does mean that we understand the world on the basis of certain of our real experiences in it, even if our understanding is metaphorical in nature, as it commonly is. Metaphorical understanding of our experience is a natural consequence of being neural beings with both bodies and brains connected as they are, with the kind of neural learning capacities that we have.

Abstract concepts don't just float in the air. They have to be given embodied meaning somehow. Embodied metaphor is a major mechanism for characterizing how we understand abstract concepts.

### Common complexes of primary metaphors

Neural binding does not just create complex schemas. It also creates complex metaphors, many of them so commonplace as go unnoticed. A Linear Scale is a Vertical Line with a schema bound to it. The schema has a Bottom, a Top, and Distances from the Bottom to points along the line. The vertical line with this schema also has a metaphor bound to it: More Is Up; Less is Down, where the verticality is in the source domain of the primary metaphor. This has a metaphorical inference, namely, Comparison of Amount Is Relative Height. Thus, *Your income is* ***higher*** *than mine* and *You have a* ***bigger*** *income than me* both mean that you make **more** money than me.

To this complex we bind the primary metaphor Change is Motion. Then, *My income* ***rose*** and *My income* ***grew*** both mean that my income changed so that I made **more** money.

Now consider the commonplace primary metaphor, Linear Scales are Paths. This primary metaphor can be seeing in expressions like *Harry is* ***way ahead of*** *Bill in athletic ability* and *Sally's intellect* ***is way beyond*** *Max's*. The metaphor is as follows:

- Source of Motion maps to Bottom of Line.
- Location along Path of Motion maps to Point on Line.
- Distance from Source of Motion maps to Height on Line.
- Being Ahead maps to Being Higher.
- Being Behind maps to Being Lower.

We now bind another metaphor to this complex: Fictive motion, or A Line Is the Motion Tracing the Line. This is the metaphor in sentences like *The road* ***runs through*** *the woods* and *The roof* ***slopes downward***. Binding this metaphor to the complex we have yields sentences like *Sally's intellect* ***goes way beyond*** *Max's* and *Corporate profits are* ***far outpacing*** *wages*, where the motion of ***go*** and **outpace** trace the distance along the vertical line.

To this complex we now bind another primary metaphor: Purposes Are Destinations, as discussed above. This has the metaphorical inference that Success Is Upward Motion and Failing is Falling. This yield sentences like *She is* ***climbing the ladder of success*** and *The middle class is* ***falling further behind*** *the one percent*. Note that ***behind*** in *falling behind* suggests forward motion, while ***falling*** suggests upward motion against a force pulling one downwards.

Suppose we now bind to the Purposes are Destinations metaphor an Impediment to Motion, namely, a Rigid Container that constrains motion out of the container. This gives rise to metaphorical sentences like *It's hard* ***to climb out of*** *poverty*, *He's* ***trapped in*** *poverty* and *She started* ***climbing the corporate ladder and hit the glass ceiling***.

This metaphor complex includes a Vertical line with a Bottom to top schema bound to it, and metaphors More Is Up, Comparison of Amount is Relative Height, Change is Motion, Linear Scales are Paths, A Line Is the Motion Tracing the Line, Purposes Are destinations, Success is Upward Motion and Failing is Falling, and Being in a Rigid Container constrains Motion Out of It. Because these are virtually all primary metaphors, we learn complexes of them with ease, without noticing all that is in the complex. Indeed, when one does notice the metaphors in the complex, a sentence like *He's trapped in poverty* may seem literal.

The Neuroscience Moral: Such complex conceptual metaphors are embodied via many different brain regions. There no single region for understanding complex ideas of any sort. Current neuroscience techniques are not likely to find evidence of all the metaphors in such a complex. Neuroscientists studying the anatomy of activation by metaphor with current techniques should probably keep to simple cases.

A General Moral: Primary metaphors—even complexes of many of them—are so natural, embodied, and deep that they can structure ones understanding without noticing that they are there. The neuroscience of concepts leads to a general principle: **You can only understand what the neural circuitry in your brain allows you to understand**. If you don't notice that you are using circuitry that is metaphorical, you will take the metaphors as being literal.

### Linguistic metaphors

Since language expresses thought, language expresses metaphorical thought as well. But in addition, grammars allow language to combine thoughts to produce an unlimited range of possible thoughts. That works for linguistic metaphor as well. Grammar allows us to combine metaphors to produce an unlimited range of new metaphorical ideas—a range that draws on primary metaphors and basic complexes of primary metaphors, but which goes way beyond those. The contemporary study of *figurative* *language* draws upon primary metaphor and complexes of primary metaphor, combining with grammar to produce that unlimited range of complex metaphorical thought (See Dancygier and Sweetser, 2014).

The simplest case of linguistic metaphor makes use of simulation in context. Imagine someone offering an explanation of something and his respondent says *That's just not clear*. In the Thought As Vision metaphor system, Understanding Is Seeing Clearly. In the context of a proposed explanation, the word *clear* activates the Thought as Vision system and the sentences metaphorically conveys that the speaker doesn't understand the explanation. The context activates the target domain of the metaphor and the language supplies the source domain.

Another simple case is a head noun preceded by a modifying adjective, as in *brilliant student*. Here the noun *student* is the target concept and the adjective *brilliant is the metaphorical source*. In the Thought as Vision system, an especially bright light source enables especially clear vision, by oneself and/or others. Metaphor simulation is needed here. A *student* is someone who is trying to understand some subject matter. If that student is a source of metaphorical light, then the student has a capacity that is a causal source of her own clear understanding. That constitutes her “brilliance.”

Sullivan points out that the adjective in such cases cannot be the target and the noun, the source. Thus, ^*^intelligent light is metaphorically ill-formed, where intelligent is the target and light is the source. However, there are cases where the adjective is target and noun is source, namely, where the adjective is a domain adjective, that is, an adjective that names a domain, as in *spiritual*, where “emotional” specifies that target domain as emotion and “intelligence” is applied from the source domain of the cognition.

A linguistically naïve view of metaphor characterizes the basic form of a metaphor as A is B (as in “the student is brilliant”), where A is the target and B is the source. But that fails in the case of domain adjectives. *Spiritual wealth* has spiritual defining the target domain and *wealth* defining the source domain. To understand *spiritual wealth*, you have to try to simulate a frame-to-frame mapping from the domain of wealth to the domain of spirituality, for example, a considerable wealth might map to considerable spirituality, multiple forms of wealth might map to multiple forms of spirituality. But the A is B form is not available for spiritual wealth. ^*^His wealth is spiritual is ill-formed in metaphorical grammar. To get some sense of the range of such cases, consider *emotional intelligence*, but not ^*^His intelligence is emotional; *economic war*, but not ^*^This war is economic.

Metaphor is woven into grammar in complex ways. A common example of metaphor in grammar is described as the construction *the X of Y*, where X is a metaphor source and Y is a metaphor target. But the real examples are more complex. Consider the following examples: *He is in* ***the grip of anger***. *We're riding in the fast lane on* ***the freeway of love***.

In the first case there are two metaphors that act together: Emotions Are Exerters of Force and Control is Control by the Hands. Anger is a special case of an emotion exerting force and thereby control, which is metaphorically control by the hands. That is what it means to be in the “grip” of anger.

In the second case, the metaphors are Love is a Journey and Action is Motion. But there is an extra wrinkle. The freeway is a metonymy; it stands for travel on a freeway. Driving in the fast lane is the specific mode of travel. It is exciting. It is reckless. You could get hurt. The sentence as a whole, with that construction and the metonymy describes a reckless love affair that is exciting but can lead to emotional harm.

#### Everyday complexity of linguistic metaphors

Metaphors play crucial roles in complex ideas. On Sunday, June 26, 2011, the following headline appeared in the main column on the front page of the NY Times. It was read by millions:

Insiders Sound an Alarm

Amid a Natural Gas Rush

Productivity of Shale Wells is a Concern —

Investor Flood Spurs Talk of Bubble

Let us look at some of the metaphors, one at a time.

***Insiders*** An institution is understood as a Container, with an inside and an outside. Those on the inside of the institution are called “insiders.” The natural gas industry is such an institution. “Insiders” have “inside information” that the institution tries to keep inside, often because the stock price would change (in this case, fall) if the true information were known “outside” the industry.

***Sound an alarm*** An alarm is a loud warning sound indicating immediate danger. To “sound” an alarm is to create a loud alarm sound heard by those in danger of being significantly harmed. In this case, the metaphorical “harm” is financial. Financial “harm” is understood as loss of money in the market.

Putting these together, we form the idea that people with “inside information” about an industry are loudly warning that investors in that industry may lose a lot of money on their stock investments.

***Amid*** Amid is a spatial term indicating that a physical entity is surrounded by a lot of other physical entities.

***Natural gas rush*** This is a metaphor based on “Gold Rush,” in which a large number of people with little real information traveled hurriedly to find gold for the taking. Some did get very rich, but most people worked very hard digging for gold without finding any. In this metaphor, what is preserved is the “rush to get rich quick.” What is changed is that natural gas has replaced gold as the way to get rich quick.

Putting all this together via bindings, we get: People working for the natural gas industry who have “inside information” find themselves surrounded by people trying to get rich quick in the natural gas industry and are warning those people of possible loss of money in natural gas stock.

Cases like this are everywhere. Just pick up a newspaper or newsmagazine and start reading. The individual metaphors contribute pieces of knowledge. To piece this knowledge together, the meanings of the individual metaphors have to be combined. That is, neural circuitry must be activated to form an overall coherent meaning, In the neural theory of language, the problem of what gets bound together neurally is called the “best fit” problem: what circuitry can be activated with the least energy to fit the pieces together? The brain is a physical system that works by conservation of energy. The stronger the synapses in a circuit, the less energy it takes to activate that circuit. That means that circuitry with the strongest existing synapses are most likely to be activated to form a best fit. In short, the brain will tend to use what it already knows as much as possible to create a “best fit.”

Neural computational modeling of “best fit” for a limited range of cases has been done by Bryant (2009).

### Complex metaphorical blends

Consider the example, “*Investor Flood Spurs Talk of Bubble*.”

The concept of “inflation” is based on an economic metaphor that real value is substance, and “inflated value” is made up partly of substance and partly of air. Real value is the ability to yield at least a certain amount of profit on an ongoing basis. Inflation occurs when the price of a stock or property gets higher than its real value. Metaphorically, the inflated part of the value is air, not substance.

The concept of a “bubble” comes with an image and knowledge about the image: the bubble is constituted of a fixed amount of substance. The bubble gets bigger when air is pumped into it. The amount of substance is fixed, most of the bubble is air with no substance, and the surface of the bubble gets thinner as the bubble gets bigger. Eventually, the surface gets so thin that bubble breaks and collapses.

In the stock market, a metaphorical bubble is a fixed amount of stock or property. As more people invest in it, the price may go up while the real value does not. That is, there is no “substance” to the investments. Eventually, the amount of value per unit of price is so little, that investors withdraw their investments, and the value *drops* precipitously (“a collapse”). The primary metaphors here are Real Value Is Substance, Inflated Value Is Air; *More Is Up*; and A Success is A Rise; A Failure is A Fall. Success in investing is a gain of real value of investments. Failure in investing means a loss of real value of investments.

In a market segment, a certain amount of investment is needed for the market segment to produce real value. Too many investors can drive stock prices up beyond real value and result in inflated value. Too much inflation produces the threat of a “bubble” that will break, and result in a considerable loss in the value of investments.

A literal flood is large body of uncontrolled rushing water that can sweep people up in it, can do a lot of damage, and harm those caught in the flood. An “investor flood” is a metaphorical flood made up of investors. This is made up of a primary metaphor, Multiplex Is Mass, in which a large number of unspecified indistinguishable individuals is conceptualized as a fluid mass, as when you see hundreds of sheep at a distance as a sheep, or when you see a lot of people as a crowd *flowing* through the streets. Investors who buy stock in a market segment are metaphorically understood as “entering” the market segment. Thus, the market segment is understood as a bounded region of space, and buying stock that is part of that market segment is seen as “entering.” A flood moves in a direction, and hence an “investor flood” refers to a large mass of investors entering a single area of the market.

The word “spur” literally refers to the spur on the boot of someone riding a horse. The rider spurs the horse to make it move by inflicting a small pain creating fear of a greater pain to come if the horse does not move. The primary metaphor here is Action Is Motion. “Spur” means to cause action by inflicting fear of pain. The second metaphor used is Financial Loss Is Painful Harm. Metaphorically, “spur” in this case means that the uncontrolled flood of investors in natural gas is causing talk of a bubble because of a fear of financial loss.

Understanding “*Investor Flood Spurs Talk of Bubble”* makes use of a number of very general metaphors: Multiplex Is Mass; More Is Up, Less Is Down; Success is Rising, Failing is Falling; Action is Motion; Financial Loss Is Pain; Real Value Is Substance, Inflation is Air. In addition certain frames are used: A flood frame, a bubble frame, and a spurring frame. From the perspective of the brain, these are neurally activated and neurally bound together in just the right way via grammar and what is called “best fit.”

Note that meaning of “*Investor Flood Spurs Talk of Bubble*” does not first assign a literal meaning to the phrase and then apply a metaphor directly to the whole. Rather pieces activate fixed and very general conceptual metaphors and frames, which are then fit together via both grammar and neural best-fit mechanisms to make the most sense in context.

This is common in poetic metaphors. Dylan Thomas wrote “Do not go gentle into that good night.” The sentence has no literal meaning. But it has a powerful metaphorical meaning since it evokes three metaphors for death. “Go” activates Death Is Departure, as in “He's left us.” “Night” activates Death is Darkness. And “Gentle” activates Life is a Struggle and Death is Giving up the Struggle. The sentence as a whole is given metaphorical meaning via these three conceptual metaphors, each applying to different words in the sentence.

But one doesn't have to look to headlines or poetry. Ordinary language also works this way. Take a sentence like, “Because he skipped steps, what he said didn't add up.” Again, the sentence has no literal meaning. Two metaphors are used. “Skipped steps” evokes Thinking Is Moving and Rational Thinking is Moving Step-by-Step. “Didn't add up” evokes Thinking Is Adding, A Thought is a number to be “counted” in the addition, and the Conclusion of an Argument Is the Sum (as in “Let me sum up”).

### Neural binding creates “blends”

To complete the picture we have given of the current state of metaphor theory, we need to consider some examples of “blends” (Fauconnier and Turner, 2002; Grady et al., 1999). During the home run race in which Mark McGuire and Sammy Sosa sought to break the home run records of Babe Ruth (60 in 154 games) and Roger Maris (61 in 162 games), the race was portrayed visually by a cartoon that appeared daily in newspapers. In the cartoon, a number of batters were lined up as in a race, with the one “ahead” on the right and the ones “behind” on the left. The text might read something like “McGuire is two games behind Ruth,” “McGuire catches up with Ruth,” and “McGuire passes Ruth.” The metaphor being used was: An attempt to break a record Is a Race between the Challengers and the Record Holder. In addition there was a neural binding. Babe Ruth played many years before McGuire and Sosa, and was long dead when McGuire and Sosa challenged his record. To allow the metaphor to apply, a neural binding is needed, identifying Ruth of yesteryear and the contemporary challengers as racers in the same race at the current time. The metaphor, plus the neural binding, creates what is called a “blend.”

Another classic example of a “blend” can be seen in cases where the following metaphor applies.

The Profession Metaphor:

A Person who performs actions with a certain *characteristic*.

Is A Member of a Profession known for *that characteristic*.

This is a metaphor, but it also has a neural binding across the source and target: the “characteristic” must be the same. The result of the metaphor plus the binding is called a “blend.” The most famous example is the pair:

1. My butcher is a surgeon.
2. My surgeon is a butcher.

These draw upon the following frame-based knowledge:

The Butcher Frame: A butcher is someone who characteristically cuts without care and control.

The Surgeon Frame: A surgeon is someone who characteristically cuts with great care and control.

The same metaphor applies in both cases, but with different “characteristics” and different professions. In (1), the source domain Profession uses the Surgeon Frame (a special case of Profession), and the “characteristic” is “cutting with great care and control.” In (2), the source domain Profession uses the Butcher Frame (a different special case of Profession) and the “characteristic” is “cutting without care and control.” The example uses three kinds of mechanism: A metaphor, a binding, and two frames that are special cases of Profession in the source domain of the metaphor.

### Metaphors apply to narratives

In any culture, there are narratives. Each narrative has certain dimensions of structure. There is a frame structure, a linear order structure, an emotion structure, and a metaphor structure. The clearest description of how these metaphorical narratives work is given in Chapter 1 of *The Political Mind* (Lakoff, 2008). The emotion structure is particularly interesting.

The Hero-Villain narrative begins with a Villain doing harm or threatening a Victim: Hearing of the harm, you feel anger or outrage. The Hero encounters the Villain and you don't know who is going to win. You feel fear or anxiety. The Hero wins. You feel relief and joy. Such “canned emotions” are built into narrative structures. Moreover, the Hero-Villain narrative can apply, via metaphor, to a political race, to scientific discovery (e.g., *The Double Helix*), to a whistle-blower at a company that is endangering the public (e.g., *Erin Brockovich*).

Jenny Lederer has analyzed a children's story from this perspective. For example, there is a story about a young fish (“The Noble Gnarble”) living at the bottom of the ocean who wants to see sunlight. The fish swims up and up, encountering a new danger at each level and overcoming the danger by virtue of what would normally be seen as a handicap that happens to be just the advantage needed to escape the danger. The story is a classical Overcoming-Obstacles-to-Reach-a-Noble-Goal narrative, applied metaphorically to a young fish. Such metaphorical narratives are everywhere.

### How do we understand really complex metaphors?

Metaphorical understanding is based on the embodiment imposed by primary metaphors, which arise via ordinary neural mechanisms when commonplace embodied experiences regularly occur together. Linguistic expressions that are metaphorical are typically complex from a conceptual point of view. They may use a number of conceptual metaphors (many of them primary metaphors) as well as frames and bindings. What are called “blends” arise from metaphors and/or frames plus neural bindings.

What neural bindings occur is often a matter of grammar plus “best fit” in context. According to our current theory of “best-fit,” complex neural circuitry is activated in context when that circuitry has the overall strongest synaptic strength in that context, and therefore can be activated with the least energy.

What is remarkable is that this is done instantly. No special talent is needed. Millions of readers read the above headlines in the NY Times, understood them instantly, and never noticed that there was anything unusual about them.

### Cascades

Narayanan and I are in the process of developing a theory of neural cascades to make sense of this data and much more. We distinguish learned cascade circuitry from a functioning cascade of activation and inhibition. Cascades are two-way circuits linking diverse brain regions connected to the body, allowing meaning from multiple realms of embodied experience to “give meaning” to linguistic, gestural, and other aspects of form. Each link in a cascade circuit does very little, but they add up to produce all of human thought.

### Embodiment: the central issue

All of what we have been discussing stress the centrality of embodiment as *the* mechanism of meaningfulness. It may be relatively obvious that sensorimotor embodiment plays a role in concepts that are not abstract, like running, kicking, seeing, smelling, and obvious concepts having to do with acting and perceiving. The neural theory of metaphor allows for the sensorimotor system to account for the meaning of abstract concepts as well, in the ways that we have seen throughout this paper.

A theory of cascades is necessary for two reasons: In complex concepts that make use of multiple primary concepts and primary metaphors, there will be a multiplicity of embodiment. Cascade theory provides the circuitry necessary to carry this out. it also provides the circuitry necessary to link the embodiment of linguistic form (in sound, writing, sign, and gesture) to the embodiment of meaning.

### Multimodality, not modularity

A major moral: From all the examples given above, it should be clear that there is no one “module” in the brain that handles language, or metaphor, or abstract thought. It takes extensive cascade circuits linking many diverse brain regions to allow for the indefinitely large variety of human reason and imagination.

### Epilog

This volume is a contribution to the scientific study of how the human brain can give rise to the details of thought and language—in this case, metaphorical thought and language. Neuroscience alone cannot answer this question, since it does not study the details of thought and language. Cognitive linguistics does. Hence, this paper. Experimental embodied cognition research also contributes scientific research on this issue. And finally, neural computation of the sort pioneered by Srini Narayanan has allowed us to model the requisite neural circuitry and neural learning mechanisms.

The very existence of this volume is testimony to the desire for cooperation across four disciplines, an integration of which is necessary to address this issue. I would like to express my gratitude to Frontiers and to the editors of this volume for taking on such a cooperative scientific enterprise.

### Conflict of interest statement

The author declares that the research was conducted in the absence of any commercial or financial relationships that could be construed as a potential conflict of interest.
